# Omics-based analysis of mitochondrial dysfunction and BBB integrity in post-COVID-19 sequelae

**DOI:** 10.1038/s41598-024-82180-6

**Published:** 2024-12-28

**Authors:** Rupal Dhariwal, Kirtan Dave, Mukul Jain

**Affiliations:** 1 Cell and Developmental Biology Laboratory, Research and Development Cell, PIMSR, Parul University, Vadodara, Gujarat 391760 India; 2 Parul Institute of Applied Sciences, Department of Life Sciences, Parul University, Vadodara, 391760 Gujarat India; 3 Bioinformatics Laboratory, Research & Development Cell, Parul University, Vadodara, Gujarat 391760 India; 4 Parul Institute of Paramedical and Health Sciences Faculty of Medicine, Parul University,, Vadodara, Gujarat-391760, India

**Keywords:** SARS-CoV-2, COVID-19, Blood–brain barrier, Neurovascular unit, hiPSC, PBMCs, Cognitive impairment, Diagnostic markers, Biochemistry, Biological techniques, Biotechnology, Cell biology, Computational biology and bioinformatics, Biomarkers

## Abstract

The SARS-CoV-2 virus that resulted in the COVID-19 pandemic has been implicated in a range of neurological issues, such as encephalopathy, stroke, and cognitive decline. Although the precise mechanism causing these issues is unknown, mounting evidence shows that blood–brain barrier (BBB) disruption is probable2 a major factor. The integrity of the blood–brain barrier (BBB), a highly selective barrier that divides the brain from the systemic circulation, is crucial for preserving normal brain function. By analysing the multi-transcriptome data, this work explores the neurological impacts of the SARS-CoV-2 virus and provides insight into the molecular mechanisms behind BBB breakdown and neurological symptoms in COVID-19 patients. The endothelial cells of BBB expresses inflammatory genes in response to the systemic inflammation induced due to SARS-CoV-2 remnants in the body. This raises the possibility that systemic inflammation brought on by SARS-CoV-2 and BBB integrity are correlated. Furthermore, the study highlights the pathways involved in oxidative stress and endothelial cell activation, revealing their role in COVID-19 passage through BBB and induction of systemic inflammation and advancement toward neurological disorders. The article showcases the evidence that mitochondrial dysfunction is a major aftermath associated with SARS-CoV-2 infection as the impaired Mitochondria leads to an accumulation of reactive oxygen species (ROS), triggering endothelial dysfunction, and leading to the passage of harmful molecules across the BBB. This study offers insightful information that may open up the possibilities for new treatment plans by targeting biomarkers specifically associated with inflammation and BBB dysfunctioning conditions.

**Supplementary Information:**

The online version contains supplementary material available at 10.1038/s41598-024-82180-6.

## Introduction

"Post-acute sequelae of SARS-CoV-2," another term for aftermath caused by SARS-CoV-2 infection, is a multi-systemic disease that develops after a severe acute respiratory syndrome coronavirus 2 (SARS-CoV-2) infection. The later effects of this infection can be seen in individuals of all ages and those who experienced different ranges of illness during the initial SARS-CoV-2 infection. However, the highest prevalence of these aftermaths occurs among people between 36 and 50 years old^1^. Several interconnected factors contribute to the development of various health issues associated with SARS-CoV-2. These factors include the presence of spike proteins in cells and tissues, dysregulated immune systems, reactivation of latent pathogens such as human herpesvirus 6 (HHV-6) and Epstein-Barr virus (EBV), changes in the microbiota and virome caused by SARS-CoV-2, autoimmune responses triggered by self-antibodies, microvascular clotting that leads to endothelial dysfunction, and disruptions in the brainstem and/or vagus nerve^2^. SARS-CoV-2 initiates inflammation and an immune response that can damage multiple organ systems and tissues by entering the central nervous system (CNS) through multiple pathways, including the bloodstream, olfactory bulb, and peripheral nervous system^3^. A substantial percentage of infection survivors have persistent symptoms following the acute stage of SARS-CoV-2 infection^4^. There is much concern regarding the risk of "Post-Acute Sequelae of SARS-CoV-2 infection" (PASC), including persistent neurological symptoms and other health problems. The World Health Organization (WHO) defines long-term COVID/post-COVID as the presence of symptoms in people with a history of probable or proven SARS-CoV-2 infection that extends for at least two months ^5^. According to Taquet M. et al., over 1.3 million COVID-19 survivors reported gradual improvements over time in mental health issues like depression, mood? and anxiety, however, the risk of cognitive impairment (brain fog), seizures, dementia, psychosis, and other neurocognitive conditions persisted even for 2 years^6^. Furthermore, recent research by Kumari A. et al., has demonstrated a strong association between COVID-19 and neurodegeneration, raising the possibility that COVID-19 could play a part in the emergence of neurodegenerative illnesses in the future^7^. Researchers have found neurological and cognitive symptoms as a major condition produced as a post-infection impact of COVID-19 along with symptoms such as visual, memory loss, cognitive decline, paresthesia, light, and noise sensitivity, loss of taste or smell (or anosmia), autonomic dysfunction, dizziness, and balance problems, often impacting activities of daily living. The research by Martínez-Salazar B. et al. reviewed the relationship between the COVID-19 and how it leads to impact and accelerates one of the pathophysiology of post-COVID i.e. neurodegeneration^8^. This article hypothesizes that SARS-CoV-2 can infect endothelial cells, leading to BBB dysfunction and leakage, speeding up the rate at which circulating viruses can enter the brain as evident by Patabendige A et al.,^9^. Previous research has reported that the brain requires controlled neuroinflammatory responses; on the contrary, uncontrolled neuroinflammation leads to the accumulation of immune cells, oxidative stress, and tissue damage to blood vessels and endothelial cells^10^. The invasion of SARS-CoV-2 triggers a cytokine storm and innate immune responses that could accelerate the onset or progression of neurodegeneration by causing peptides to self-assemble into toxic amyloid clumps and releasing cytokines as a result of hyperactivated microglia cells, which in turn promotes cognitive dysfunction and impaired neurogenesis^11^. Findings from articles addressing post-COVID consequences such as Davis et al., show decreased natural killer cell function, T cell exhaustion and other abnormalities in T cells, mitochondrial dysfunction, and vascular and endothelial abnormalities, including deformed red blood cells and reduced blood volume, have all been linked to this neuroinflammatory phenomenon^12^.

**Fig. 1:**
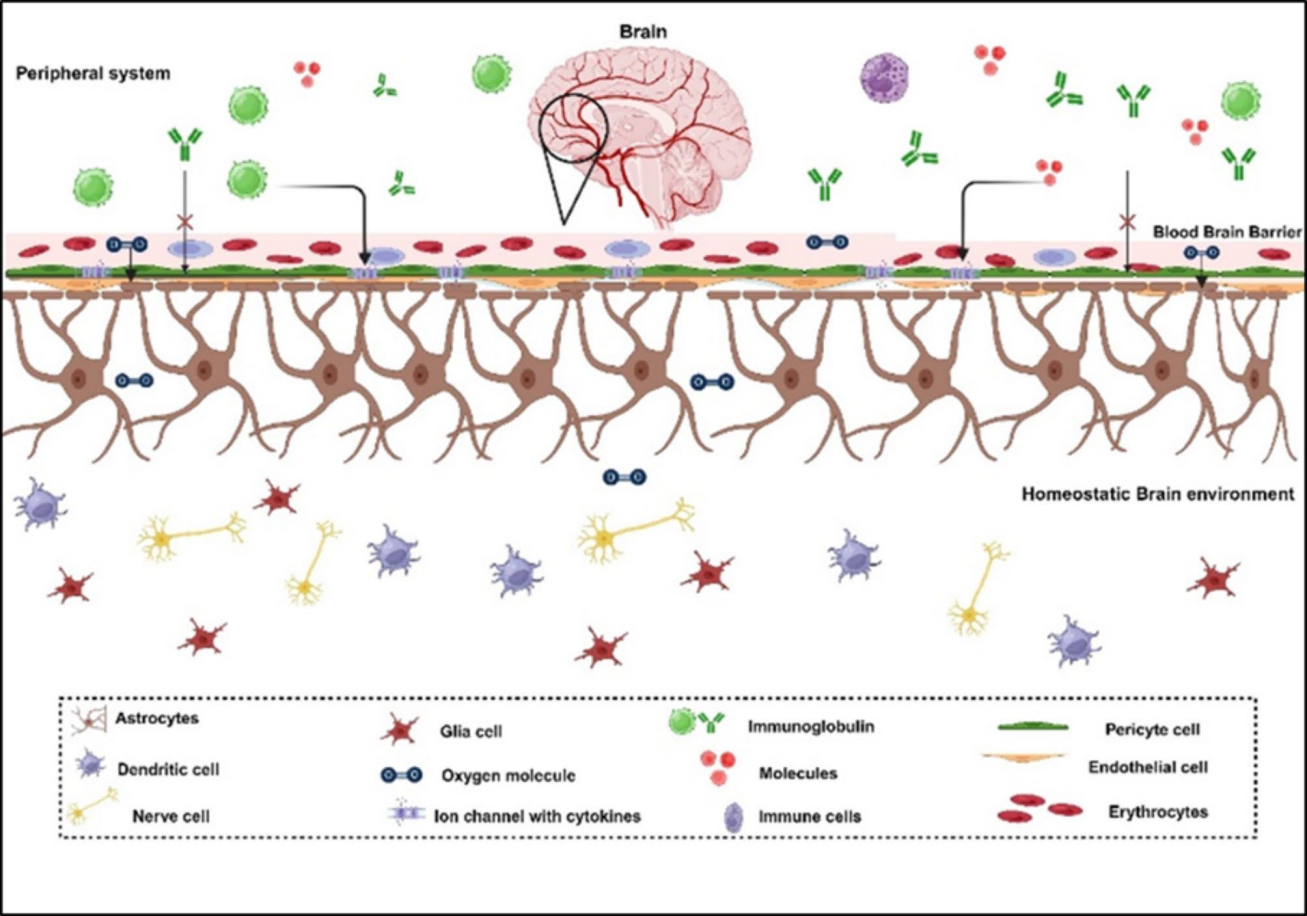
The Blood–brain Barrier acts as an insulating layer protecting the brain from direct contact with infectious agents, mediating the selective transfer of molecules and ions through channels and transporters only.

As shown in the above Fig. 1, the neurons that form a part of the neurovascular unit (NVU) modulate the BBB permeability. These capability of the NVUs to alter the BBB integrity and functionality leads to the presence of a cluster of differentially functioning cells that consists of Endothelial cells (ECs), neurons, pericytes, astrocytes, and microglia^13^. The ECs form the walls of blood vessels and capillaries and regulate BBB permeability by forming an extracellular matrix that forms tight junctions. The second cell type i.e. pericytes present at the capillary floor maintains the integrity of this extracellular matrix and tight junctions^14^. Apart from these, the neurons and astrocytes also regulate the tight junction communication and molecular transport in response to water and ion balance, regulating a wide array of chemokines and adhesion molecules. These regulatory units are dysfunctional during and in post-COVID scenarios, imposing a possible BBB disruption event happening in the body with major implications of endothelial cell shrinkage or disintegration and altered paracellular transport of molecules due to the disintegration of tight junction proteins and tight junction translocation. Multiple organ dysfunction, both structural and functional, such as cardiovascular, neurological, mental, hematological, pulmonary, and dermatological injury, is a hallmark of post-COVID-19 disorders. Numerous research directions have proposed that the neurological symptoms of post-COVID infection are caused by the blood–brain barrier (BBB) breaking down and serum components and cytokines then entering the brain^15^. Recent data suggests that one or more persistent medical conditions such as brain fogginess, difficulty concentrating, insomnia, breathlessness, muscle ache, joint pain, headache, cough, chest tightness, loss of smell/taste, cardiovascular, thrombotic and cerebrovascular disease, depression and anxiety, etc. are present in about 80% of COVID-19 infections^16^. Angiotensin-converting enzyme 2 (ACE2) receptors are found throughout the body in the brain, gastrointestinal tract, lung, heart, liver, kidney, spleen, and mucosa of the mouth and nose and allow SARS-CoV-2 to enter cells. Post COVID impacts the lungs the most since, based on CT scans, even asymptomatic COVID-19 individuals had severe lung damage. Several kinds of literature such as Raveendran et al., have characterized Post-COVID into different categories depending upon the predominant residual symptoms as post COVID cardio-respiratory syndrome, post COVID fatigue syndrome and post COVID neuro-psychiatric syndrome^17^. The respiratory system being the main entry source for the COVID infection with abundance of ACE2 receptors in the nasal lining, places the Post-COVID respiratory issues at the top priority. However, brain wellness is also a matter of much concern as even evident from major questionnaire-based surveys and research^18,19^. The article Pandharipande P. et al., reported abnormalities on lung CT, abnormal pulmonary function tests, generalized symptoms, psychiatric symptoms, and neurological symptoms mainly cognitive deficits and memory impairment^20^. Over the past few decades, there has been a sharp rise in cognitive impairments linked to the aging population as reported by Alzheimer’s Disease International (ADI) report. In 2015, 46.8 million people worldwide were estimated to be suffering from dementia. This number is expected to increase to approximately 74.5 million by 2030 and 131.5 million by 2050^21^. The prevalence of mild cognitive impairment (MCI) is 12–18% among adults over 65 years of age, and the annual progression rates from MCI to AD are 10–15%. Along with these disorders, subjective cognitive decline is a precondition for MCI and may be a sign of dementia that follows COVID-19 problems. Thus, it is crucial to identify whether post-COVID-19 complications and syndrome will put the survivors at risk for cognitive decline or any mental health issue at a rapid pace. Subjective self-reported surveys and evaluations of cognitive functioning have revealed persistent cognitive impairment and abnormalities following SARS-CoV-2 infection compared to those without illness. The discovery of higher inflammatory markers in the CSF of patients with post-COVID-19 neurocognitive symptoms, Dąbrowska E. et al., suggested that persistent immune activation and inflammation in the CNS may contribute to neurocognitive manifestations of post-COVID^22^. This article thus aims to analyze the inflammatory markers released and present in the periphery system due to systemic inflammation that persists even after COVID-19 infection. The article will also summarize the pivotal linkage of inflammation with BBB dysregulation and the interrelatedness in progressing the post-COVID condition.

## Results

### Comparative transcriptomic analysis using DESeq2: identification of differentially expressed genes in GSE179923 and GSE251849

**Fig. 2:**
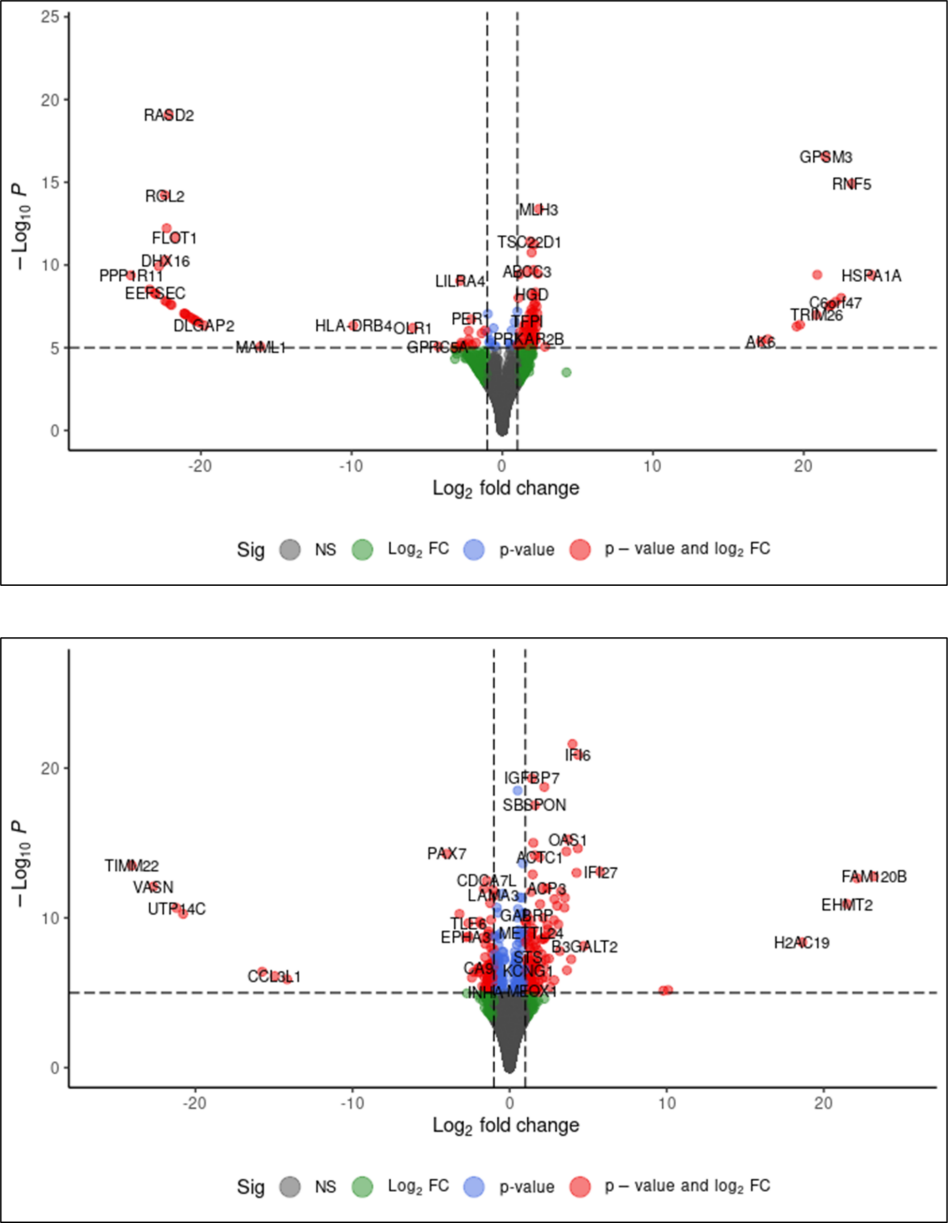
(**a**, **b**) Volcano plots representing differentially expressed genes (DEGs) between GSE251849 (n = 23) and GSE179923 (n = 30). The red points show up-regulated genes (log2 FC ≥ 0.5 and adjusted p-value < 0.05). The x-axis shows the log2 fold change, indicating the level of gene expression differences, while the y-axis indicates the statistical significance (-log10 P-value). Genes such as GABRP, CCL3, etc. that are significantly upregulated are marked in red, and genes such as LAMA3, STS, etc. that are downregulated are shown in blue. Threshold lines represent the cut-off of 0.05 for both statistical significance.

The transcriptome data of NGS was compared with the human index differential expression between COVID-infected mock infection and the numbers of significant DE transcript clusters produced were compared in Fig. 2**.** The analysis performed with DESeq2 with adjusted p-value < 0.05 on R Studio returned with different values. The analysis of GSE179923 and GSE251849 demonstrates a significant quantity of genes exhibiting modified expression levels under the given circumstances. The study GSE179923 identified 1760 genes that were upregulated and 1424 genes that were downregulated. In study GSE251849, 555 genes were found to be upregulated and 799 genes were found to be downregulated. Both studies met the criterion of having an adjusted p-value of less than 0.05 and log-fold change thresholds.

### Analysis of the pathways associated with significantly dysregulated transcriptome data of both the datasets

**Fig. 3:**
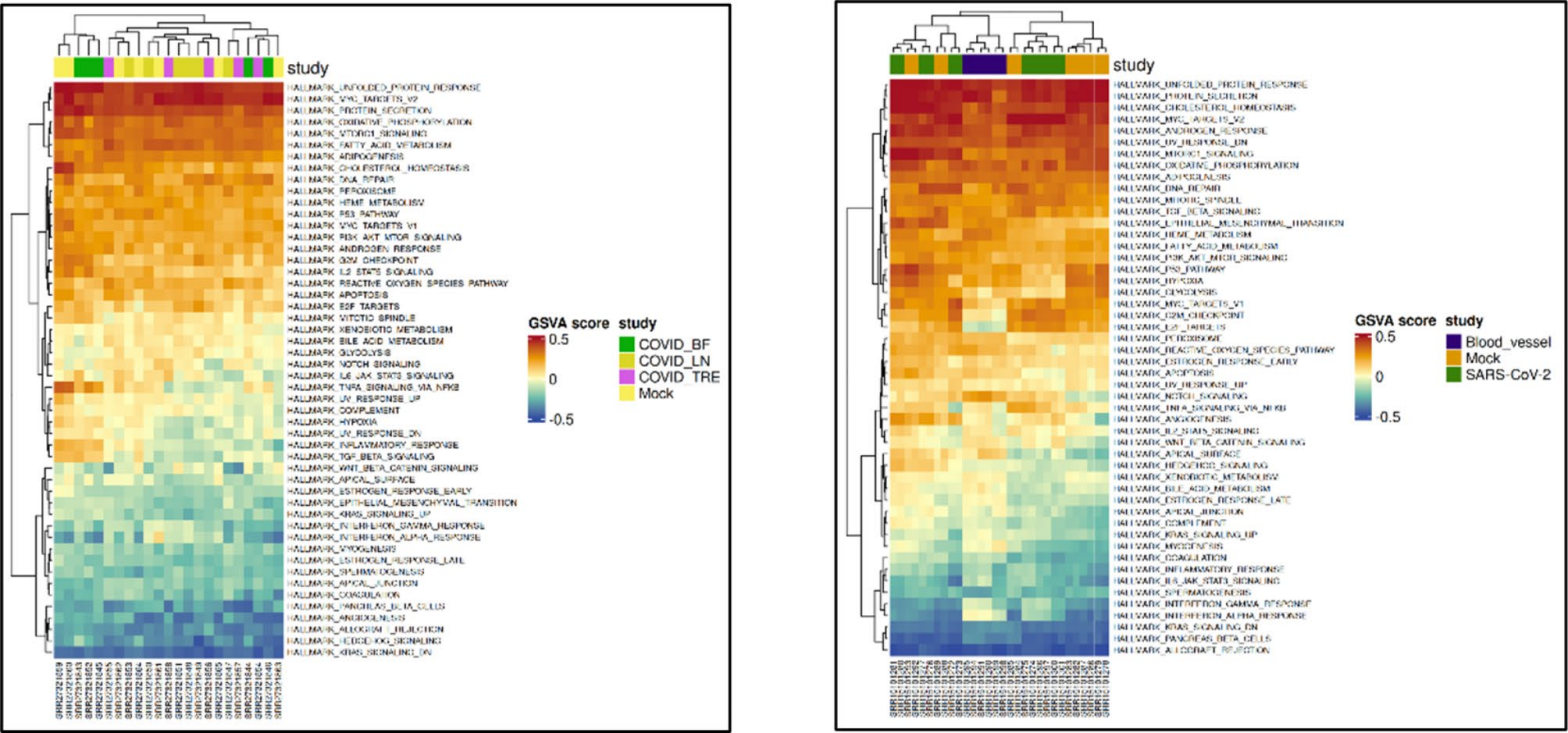
Heat map analysis of the pathways affected and dysregulated by the dysregulated gene sets from both datasets.

The Fig. 3 predicts the effect of dysregulated genes in different sample class of both the datasets. It is evident that the pathways such as oxidative phosphorylation, inflammation associated pathways, ROS generation pathways, etc. indicates a close association between the transcriptome being dysregulated and their effect on this crucial metabolic pathway. The dysregulation in these pathways will link in promoting dysfunction of brain health and associated parameters as the mentioned pathways have prominent role in maintaining the normal brain functioning and homeostasis by regulating the protein clearance, free radicle removal and coordinating synaptic functioning.

### Role of significantly dysregulated genes in major pathways in the human system

**Fig. 4:**
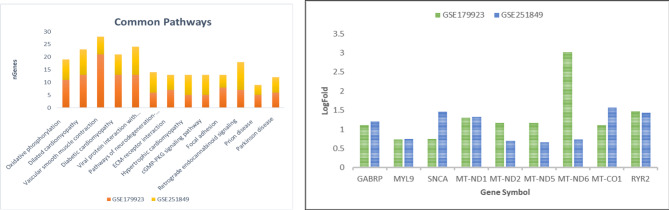
(**a**) GO Pathway analysis with highly dysregulated gene set (**b**) Expression analysis of top-most dysregulated gene sets from within the two datasets.

The GO analysis in Fig. 4a of up-regulated genes such as MT-ND1, MT-ND2, MT-ND3, MT-ND5, MT-ND6, ITGA2B, ITGB3, ITGB5, and many other as mentioned in Table 1 reveals the potential role of pathways in BBB disruption. The pathways associated with genes in Fig. 3 highlight multiple mechanisms involved in maintaining the blood–brain barrier’s (BBB) structural integrity. These mechanisms include oxidative phosphorylation and reactive oxygen species (ROS) pathways, which play crucial roles in cellular metabolism and oxidative stress. Oxidative phosphorylation, the main cellular process for generating ATP, is crucial for maintaining the integrity of the blood–brain barrier (BBB) by powering energy-dependent processes like BBB maintenance^23^. Dysfunction in this pathway can lead to cellular stress and damage from reactive oxygen species (ROS), further compromising the BBB. Hypertrophic cardiomyopathy reduces blood flow to the brain, limiting oxygen supply and worsening oxidative stress. This can trigger inflammatory responses that weaken the BBB^24^. The cGMP-PKG signaling pathway regulates BBB function by influencing blood vessel tone and permeability. Disruptions in this pathway can weaken the BBB by affecting endothelial barrier function, another contributing factor to BBB dysfunction^25^. Endocannabinoid signaling plays a crucial role in regulating neuroinflammation and vascular function. Disruption of this signaling pathway can compromise the integrity of the blood–brain barrier (BBB) and affect the neurovascular unit^26^. Prion diseases are marked by the accumulation of misfolded proteins, which induces inflammation and oxidative stress within the brain. This process undermines the BBB, increasing its permeability and facilitating disease progression. Similarly, Parkinson’s disease is associated with inflammation and oxidative stress, characterized by the aggregation of alpha-synuclein, which further impairs BBB integrity and exacerbates neuronal degeneration. These mechanisms disrupt the BBB through oxidative stress, inflammation, and damage to endothelial cells. Notably, in both studies, nine genes—GABRP, MYL9, SNCA, MT-ND1, MT-ND2, MT-ND5, MT-ND6, MT-CO1, and RYR2—are upregulated as illustrated in Fig. 4.

**Table 1:** List of dysregulated genes among the two datasets.

Pathway name	GSE179923	GSE251849
Oxidative Phosphorylation	MT-ATP6 MT-ATP8 MT-CO1 MT-CO2 MT-CO3 MT-CYB MT-ND1 MT-ND2 MT-ND3 MT-ND5 MT-ND6	MT-CO1 MT-ND1 MT-ND2 MT-ND5 MT-ND6
cGMP PKG signalling pathway	MYL9 ADRA1D EDNRB MYH6 PDE3A PLN MYLK3	MYL9 ADCY6 GUCY1A1 GUCY1B1 MYLK SLC8A3 TRPC6 PDE5A
Viral protein interaction with cytokine and cytokine receptor	XCR1 CXCL8 CCL2 TNFSF14 CXCL14	PF4 PF4V1 PPBP CXCL5
Vascular smooth muscle contraction	MYL9 ADRA1D EDN3 PLA2G2A ACTG2 MYLK3	RAMP1 MYL9 ADCY6 GUCY1A1 GUCY1B1 MYLK CALD1 PPP1R14A
Focal adhesion	MYL9 COL2A1 COL4A1 COL6A6 MYL7 BCL2 SPP1 MYLK3	MYL9 EGF PARVB ITGA2 ITGA2B ITGB3 ITGB5 MYLK VEGFC VWF
ECM receptor interaction	COL2A1 COL4A1 COL6A6 FREM2 SPP1	GP1BA GP1BB GP9 ITGA2 ITGA2B ITGB3 ITGB5 VWF
Retrograde endocannabinoid signaling	CNR1 GABRP MT-ND1 MT-ND2 MT-ND3 MT-ND5 MT-ND6	ADCY6 GABRP GNAO1 GNG11 MT-ND1 MT-ND2 MT-ND5 MT-ND6
Parkinson disease	LRRK2 MT-ATP6 MT-ATP8 MT-CO1 MT-CO2 MT-CO3 MT-CYB MT-ND1 MT-ND2 MT-ND3 MT-ND5 MT-ND6 SNCA	MT-CO1 MT-ND1 MT-ND2 MT-ND5 MT-ND6 TUBA8 SEPTIN5 BCL2L1 SLC18A2 SNCA TUBB1
Prion disease	HSPA8 MT-ATP6 MT-ATP8 MT-CO1 MT-CO2 MT-CO3 MT-CYB MT-ND1 MT-ND2 MT-ND3 MT-ND5 MT-ND6 RYR2	MT-CO1 MT-ND1 MT-ND2 MT-ND5 MT-ND6 TUBA8 RYR2 TUBB1
Pathways of neurodegeneration multiple diseases	CHRM1 LRRK2 MT-ATP6 MT-ATP8 MT-CO1 MT-CO2 MT-CO3 MT-CYB MT-ND1 MT-ND2 MT-ND3 MT-ND5 MT-ND6 BCL2 RYR2 SNCA WNT2 WNT6 WNT10A WNT5B FZD8	MT-CO1 MT-ND1 MT-ND2 MT-ND5 MT-ND6 TUBA8 SEPTIN5 BCL2L1 RYR2 SNCA TUBB1
Hypertrophic cardiomyopathy	MYH6 RYR2 ACTC1 TNNT2 TTN	ITGA2 ITGA2B ITGB3 ITGB5 RYR2 SLC8A3
Dilated cardiomyopathy	MYH6 PLN RYR2 ACTC1 TNNT2 TTN	ADCY6 ITGA2 ITGA2B ITGB3 ITGB5 RYR2 SLC8A3
Diabetic cardiomyopathy	MT-ATP6 MT-ATP8 MT-CO1 MT-CO2 MT-CO3 MT-CYB MT-ND1 MT-ND2 MT-ND3 MT-ND5 MT-ND6 PLN RYR2	MT-CO1 MT-ND1 MT-ND2 MT-ND5 MT-ND6 RYR2

These genes play a crucial role in maintaining BBB functionality. Gamma-Aminobutyric Acid Type A Receptor Pi Subunit, or GABRP, is a part of the GABA-A receptor that is very important for stopping neurotransmission. Changes in GABA signalling can affect the permeability of the blood–brain barrier (BBB) by affecting the function of endothelial cells and the integrity of tight junctions. Dysregulation of GABRP can also lead to the breakdown of the BBB and increased permeability^27^. MYL9 (Myosin Light Chain 9) is involved in regulating smooth muscle contraction, and its dysregulation can impact vascular smooth muscle function, altering blood flow and pressure. This compromises endothelial cells and tight junctions, affecting BBB integrity. SNCA (Alpha-Synuclein), which is linked to neurodegenerative diseases like Parkinson’s, can build up and cause neuroinflammation and oxidative stress, which damages the integrity of the BBB. Misfolded alpha-synuclein can directly damage endothelial cells and tight junction proteins, increasing BBB permeability. The mitochondrial genes MT-ND1, MT-ND2, MT-ND5, and MT-ND6 encode subunits of Complex I of the mitochondrial respiratory chain. Mutations or dysfunctions in these genes impair oxidative phosphorylation, increasing reactive oxygen species (ROS) production^28^. Elevated oxidative stress can damage endothelial cells, compromise tight junctions, and increase BBB permeability. Similarly, MT-CO1 (Mitochondrially Encoded Cytochrome C Oxidase I) creates a part of Complex IV in the mitochondrial respiratory chain. Its dysfunctioning can stop mitochondrial respiration, raises oxidative stress, damages endothelial cells, and compromises the integrity of the blood–brain barrier (BBB). RYR2 (Ryanodine Receptor 2) is involved in calcium release from the endoplasmic reticulum in muscle cells. Dysregulation of RYR2 alters calcium homeostasis, affecting vascular smooth muscle function and endothelial cell signaling, which can disrupt tight junctions and increase BBB permeability. these genes play crucial roles in BBB dysregulation through mechanisms involving oxidative stress, inflammation, endothelial cell damage, and disruptions in cellular signaling and energy metabolism, compromising BBB structural integrity and function and leading to increased permeability and potential neurological consequences.

## Mitochondrial dysfunction and blood–brain barrier breakdown

The genes listed are crucial for mitochondrial function and serve as fundamental elements of the oxidative phosphorylation pathway, which is vital for cellular energy generation. MT-ATP8 and MT-ATP6 genes are responsible for encoding subunits of ATP synthase, which are crucial for generating ATP. Disruption of these genes can lead to a deficiency in energy, thereby compromising the integrity of the blood–brain barrier (BBB). The genes MT-CO1, MT-CO2, and MT-CO3 encode subunits of cytochrome c oxidase, which play a role in the latter steps of mitochondrial respiration. The dysfunctioning of these genes might lead to a decrease in ATP production and an increase in oxidative stress. This can potentially harm the blood–brain barrier.


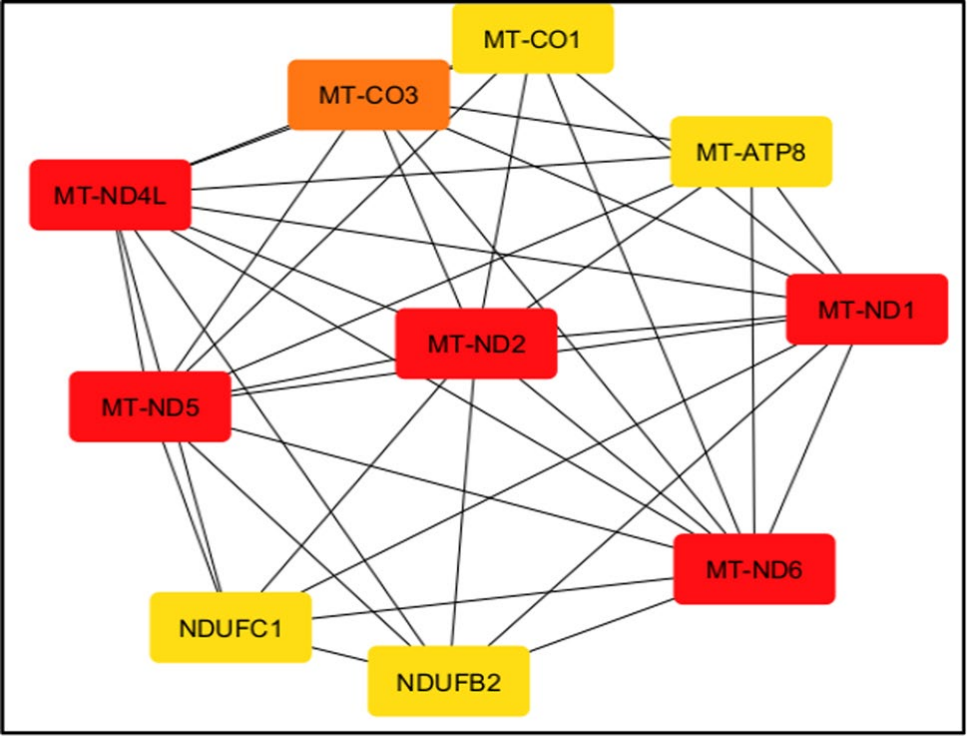


The genes MT-ND1, MT-ND2, MT-ND4L, MT-ND5, and MT-ND6 are responsible for encoding subunits of NADH dehydrogenase, which is also known as complex I^29^. If these genes do not function properly, it can lead to a decrease in ATP production and an increase in reactive oxygen species (ROS). Both of these effects are associated with the disruption of the blood–brain barrier (BBB) and the occurrence of neuroinflammation. NDUFB2 and NDUFC1, which are components of complex I, play a role in mitochondrial activity. Their dysfunctioning can worsen energy deficiencies and oxidative stress, which in turn affects the integrity of the blood–brain barrier^30^. In addition, abnormal angiogenesis leads to the development of disorganized new blood vessels, which further compromises the integrity of the blood–brain barrier (BBB). In general, although these mitochondrial genes do not have a direct role in regulating the blood–brain barrier (BBB), their malfunction can result in oxidative stress, lack of energy, and neuroinflammation, all of which can have a negative impact on the integrity of the BBB.

## Discussion

The current research aims to identify the significant association between the pathophysiology of neurodegeneration and post-COVID-19 conditions. After the course of COVID-19 infections in the human population, it has been found that recovered patients complain of brain fogginess and other mental health-related issues^31^. The pathophysiology of these conditions is linked with dysregulation of pathways such as oxidative phosphorylation, inflammatory processes, and extracellular matrix-related mechanisms. One notable factor contributing to the neurodegenerative effects seen post-COVID-19 is the disruption of the blood–brain barrier (BBB). Inflammatory mediators induced by SARS-CoV-2 infection are important causative factors for BBB dysfunction, which facilitates the entry of peripheral immune cells and other neurotoxic agents into the brain. Although no specific proinflammatory cytokines were identified as upregulated in the current study, previous studies have shown that elevated levels of cytokines such as IL-6, TNF-α, and IL-1β during SARS-CoV-2 infection can compromise BBB integrity. Major cellular compartment organizational changes caused by viral infections comprise the fragmentation of mitochondria with the generation of reactive oxygen species, interaction with the ER and Golgi apparatus, and mitochondrial perinuclear clustering. Saltzman LY. et al., have reported the upregulation of genes such as ITGB5, CASP4, BCL2, RYR2, MT-ND, and MT-CO in hindering the normal functioning of the above-mentioned pathways and eventually affects the host’s immunity and reaction to viral infection^32^. It has been studied that the coronavirus genome contains distinct mitochondrial localization signals within the 5′- and 3′-untranslated regions^33^.

**Fig. 5:**
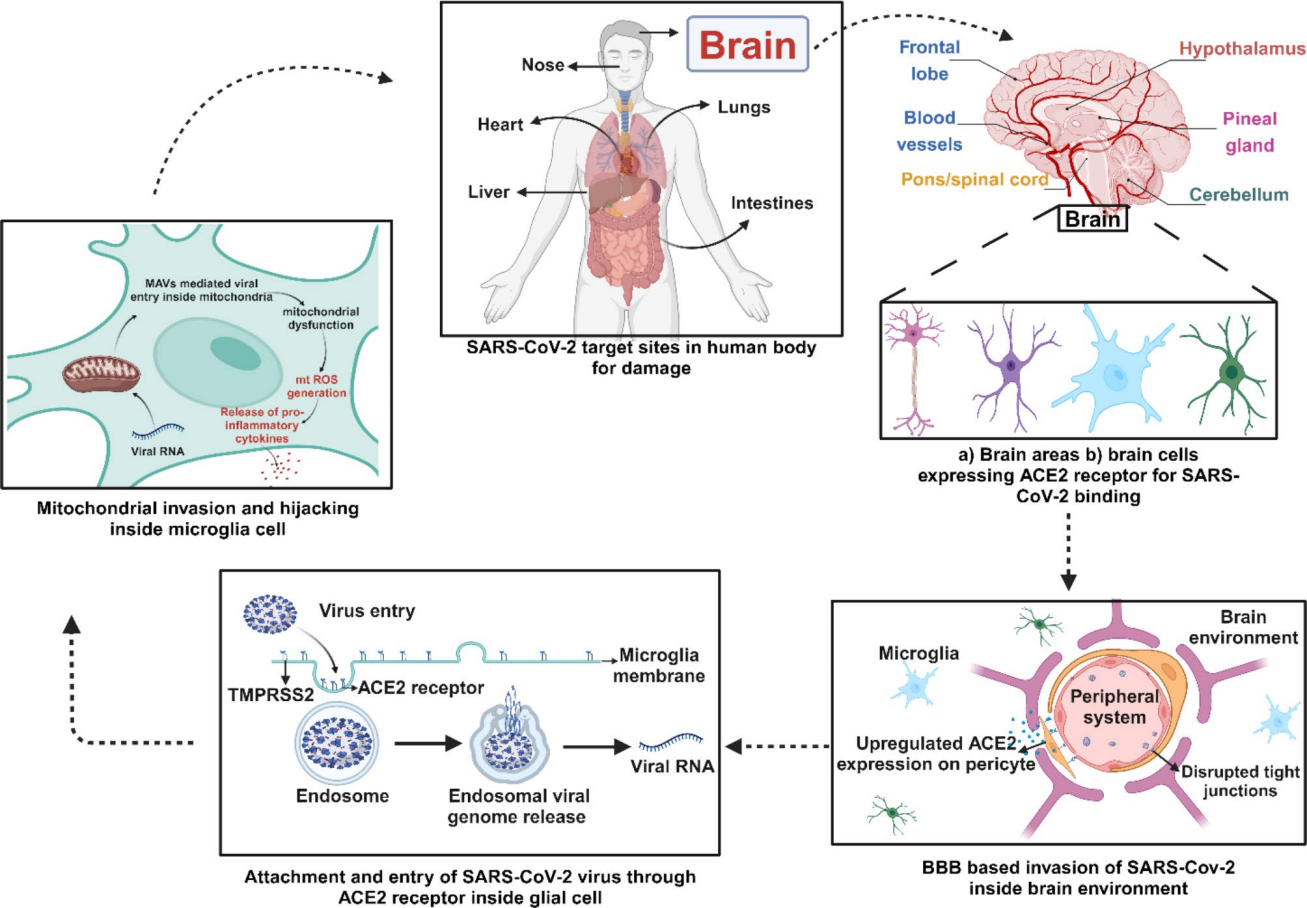
ACE2 receptor-mediated SARS-CoV-2 entry inside the human body. The expression of Angiotensin-converting enzyme 2 (ACE2) by brain region and cells facilitates the entry of viral spike protein from the peripheral system to the central nervous system. The uptake of virus genome content by the host cell results in targeted degeneration of mitochondria i.e. the powerhouse of the cell. This results in the collapse of major metabolisms of cells ultimately, causing cell death**.**

**Fig. 6:**
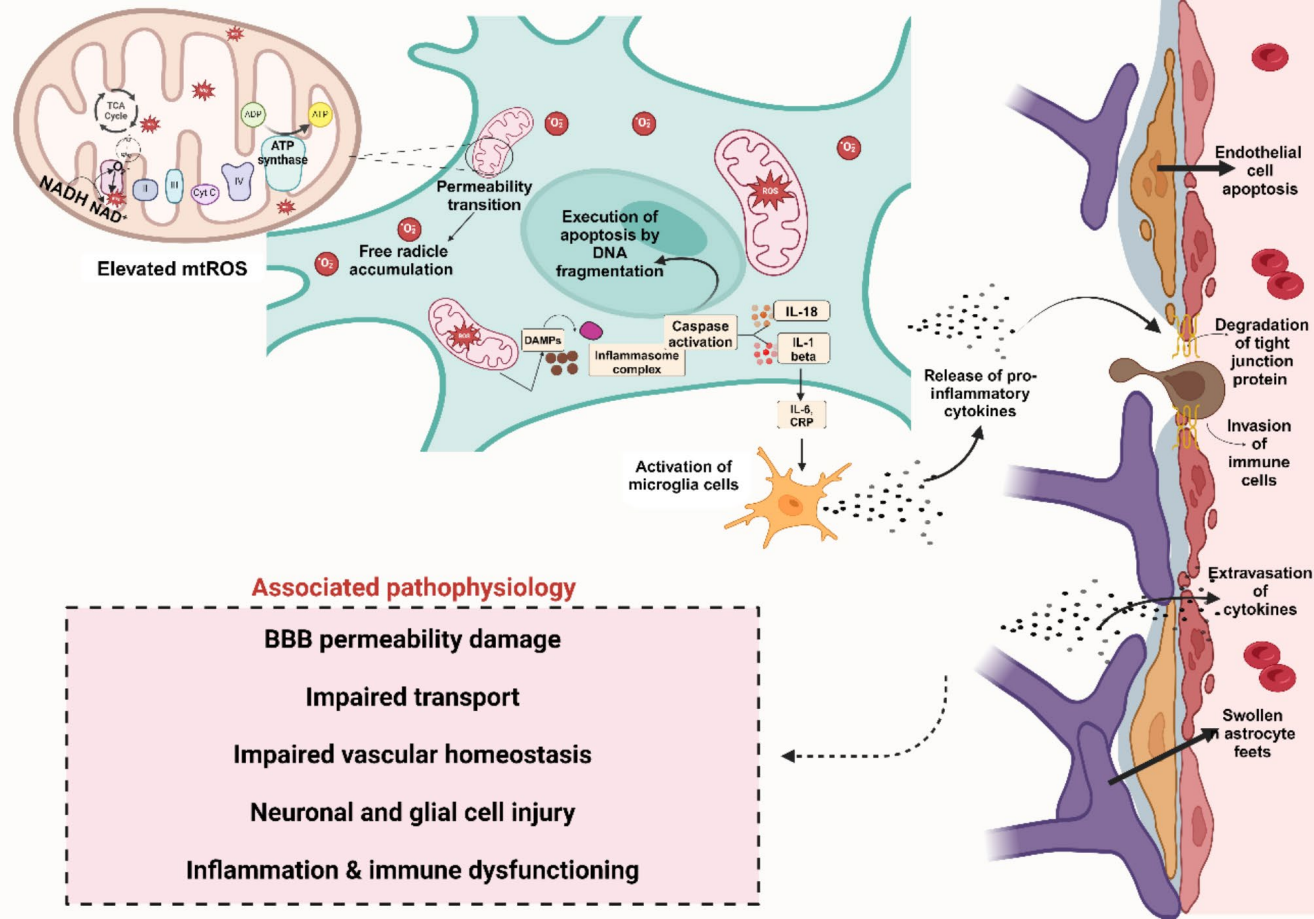
The dysfunction of mitochondrial-associated genes harms normal brain homeostasis by targeting the Oxidative phosphorylation mechanism that results in uncontrollable ROS generation. The activation of the Caspase cascade due to ROS helps produce pro-inflammatory markers that activate the resting microglia cells. The cytokines induce BBB damage by targeting the tight junction proteins and membrane integrity, causing cortical neuron death, cerebral ischemia, arrhythmia, epilepsy episodes, and also cognitive impairment. Lacampagne A et al.,^43^ have reported Ryr with a causative linkage to Alzheimer’s disease. The leaky RyR2 has been considered as the “biochemical signature” and was associated with an ER Ca^2+^ leak, through increased RyR2 open probability and a loss of memory^43^. For analysing and accessing the crucial role of the BBB proteins holding and maintaining its integrity, Table 2 has been shown below that signifies the importance of different tight junction proteins that helps in the maintenance of an isolated system enclosing the BBB. The primary goal of this research article is thus, to highlight and associate the leaky BBB during COVID infection could lead to prolonged cytokine storm and systemic inflammation thereby, triggering the brain cells to leverage a deleterious effect caused by oxidative stress that could damage a variety of cells such as BMVECs, pericytes, and astrocytes, destroying the BBB.

As evident from Fig. 5, the coronavirus localizes into host mitochondria by binding to the outer mitochondrial membrane and increases the double-membrane vesicle generation number for generating a vehicle for evasion and hiding within it infecting the body by suppressing the innate immunity through the mitochondrial functioning, calcium and iron homeostasis, steroid synthesis, apoptosis, and mitochondrial antiviral signaling protein dysregulation. Wu P. et al., carried out research with the previous generations of positive single-stranded RNA (ssRNA) coronavirus (SARS-CoV) infected Blood PBMCs through q-PCR and electron microscopy proving the higher level expression of oxidative stress, intense upregulations of stress response protein, etc.^34^. This theory was later confirmed by Shao H. et al., who found higher expression of mitochondrial dysfunction-associated genes that affect stress response, immunoregulation, apoptosis, and inflammation through microarray and q-PCR of human blood PBMCs and SARS-CoV infected cell lines^35^. Eventually, the current era of research has used transcriptome sequencing to analyze the dysfunction in SARS-CoV-infected patients. Analyzing the relevant data taken from^36,37^, our work used bioinformatics and omics tools to found the evident role of these genes in increasing ROS and inducing inflammation. Generation of mt ROS mainly takes place at the ETC, located on the Intracellular Matrix Membrane, with the major sites of the respiratory chain Complex I (NADH dehydrogenase), Complex III (ubiquinol-cytochrome c reductase), and also the dihydrolipoamide dehydrogenase enzyme. ATP, cardiolipin, mt DNA, mt ROS, and mitochondrial Ca2+ are all reverted in the cytosol or extracellular milieu during this mito-inflammation, which causes the production and distribution of a variety of pro-inflammatory mediators. This mt ROS may directly act into the organelle, promoting oxidative damage to intra-organelle molecules and mt DNA and activating pro-inflammatory signaling pathways. This mechanistic release of reactive oxygen species via activation of the caspase pathway damages and breaks the extra-cellular matrix in the BBB. Caspase-1 being the core component of the inflammasome complexes regulates the activation, production, and secretion of the pro-inflammatory cytokines IL-1β and IL-18, etc.^38^. Krasemann S. et al., have reported that the activation of inflammasome markedly decreased the expression of tight and adherent junction proteins^39^. This proves the fact that mitochondrial dysfunction will also target the BBB as evidenced below described in Fig. 6.

**Table 2:** Role of regulatory tight junction proteins managing the BBB permeability cascade.

Protein	Role in BBB permeability	Reference
Claudin	Prevents para cellular permeability	^44^
Zonula Occludens-1	Links transmembrane tight junction proteins to the actin cytoskeleton	^45^
Junctional Adhesion Molecule-A	Regulates monocyte migration and leukocyte adhesion	^46^
P-glycoprotein	Functions as an efflux pump to remove endotoxins out of brain	^47^
Glucose Transporter 1	Maintains the glucose energy requirement of the brain cells	^48^
Aquaporin-4	Controls brain water homeostasis	^49^
Caveolin-1	serves as a mediator in drug delivery through the blood–brain barrier	^50^
Transferrin Receptor	Mediates iron transport across the BBB	^51^
N-cadherin	supports the interaction between endothelial cells at the BBB	^52^

The dysfunction of mitochondrial-associated genes harms normal brain homeostasis by targeting the Oxidative phosphorylation mechanism that generates uncontrollable ROS. The activation of the Caspase cascade due to ROS helps produce pro-inflammatory markers that activate the resting microglia cells. The cytokines induce BBB damage by targeting the tight junction proteins and membrane integrity. Certain articles have shed light on how the SARS-CoV-2 induces the tight junction & integrity proteins’ downregulation leading to loss of BBB permeability. Article^40^ has reported that SARS-CoV-2 damaged the integrity of the BBB by negatively regulating the expression of TJ proteins, such as TJP1, OCDN, Itgb5, and CLDN5. It demonstrated that SARS-CoV-2 invasion of the brain increased the BBB permeability by downregulating and redistributing TJ proteins. This article also found the upregulation of pro-inflammatory markers due to inflammatory response. However, our work predicted the dysfunction of BBB permeability-related proteins, with lesser findings on the pro-inflammatory markers/cytokines molecules. This could be evident from the previous findings suggesting that the BBB breakdown or dysfunctioning doesn’t immediately result in the upregulation of pro-inflammatory cytokines. Work from Obermeier B. et al., states the progressive and gradual release and increase in the pro-inflammatory molecules as in the initial phase, the immune-modulating system could counteract the dysregulation^41^. Similarly, Prinz et al., suggest that the increase in pro-inflammatory cytokines is not solely dependent on the BBB permeability. Nevertheless, the activation of microglia cells via specific signaling would activate and promote the pro-inflammatory molecules release^42^.

Once the microglia system gets activated, the BBB integrity genes such as Integrin beta 5 (ITGB5) that bind to the extracellular matrix (ECM) and participate in cell survival, proliferation, and migration and RyR get dysregulated affecting each other’s functioning. The leakage of BBB will promote the passage and migration of inflammatory cytokines, and immune cells causing RyR receptor dysfunction. The Ryr, expressed in the cerebral cortex and dental gyrus of the hippocampus increases the Ca^2+^entry,

Our findings also highlight the role of mitochondrial genes such as *MT-CO1, MT-CO2, MT-ND1, MT-ND2, MT-ND5,* and *MT-ND6* in contributing to oxidative stress pathways^53^. The dysregulation of these genes can lead to an increased production of reactive oxygen species (ROS), which indirectly boosts pro-inflammatory cytokine activity^54^. ROS can initiate signaling cascades, such as NF-κB^55^, that facilitate cytokine synthesis. Furthermore, pro-inflammatory cytokine activity has been associated with the transcription factor BATF (Basic leucine zipper transcriptional factor ATF-like)^56^, which modulates genes involved in immune responses, including cytokine synthesis.

## Material and methods

### Data collection

The datasets analysed in this study were obtained from https://www.ncbi.nlm.nih.gov/geo/. ncbi.nlm.nih.gov/geo/. To identify the differentially expressed genes in normal and SARS-CoV-2 infection in BBB samples, we searched the gene expression omnibus (GEO) databases with terms such as “SARS-CoV-2 infection” and” blood–brain barrier (BBB)”. The database containing transcriptome profiling of normal/mock and blood–brain barrier of the COVID-19 infection was selected for further analysis. RNA-Seq data of 76 SRR files (26 cases, 12 controls) from three tissues Primary brain capillary endothelial cells (BCECs), Blood vessels, and Peripheral blood mononuclear cells (PBMCs) were collected from the Gene Expression Omnibus database (GEO), as depicted in Supplementary Table 1. The cases were infected with SARS-CoV-2 and the controls were not. The GEO accession ID for BBB and COVID-19 are GSE251849 and GSE179923. As per the details mentioned in the GEO repository, The GSE179923 dataset involves human brain-capillary endothelial cells (BCECs) derived from induced pluripotent stem cells (hiPSCs). The cells were either mock-infected or exposed to SARS-CoV-2 to study the virus’s impact on the blood–brain barrier. Similarly, the study of dataset GSE251849 focuses on patients suffering from cognitive issues linked to post-COVID, often referred to as "brain fog." The data focused on proving the fact that sustained systemic inflammation and persistent localized BBB dysfunction are the key features of Long COVID-associated brain fog. For the same purpose, the article included unaffected controls, patients recovered from SARS-CoV-2 infection, and long COVID patients with or without cognitive impairment.

### Bioinformatics analyses

**Fig. 7:**
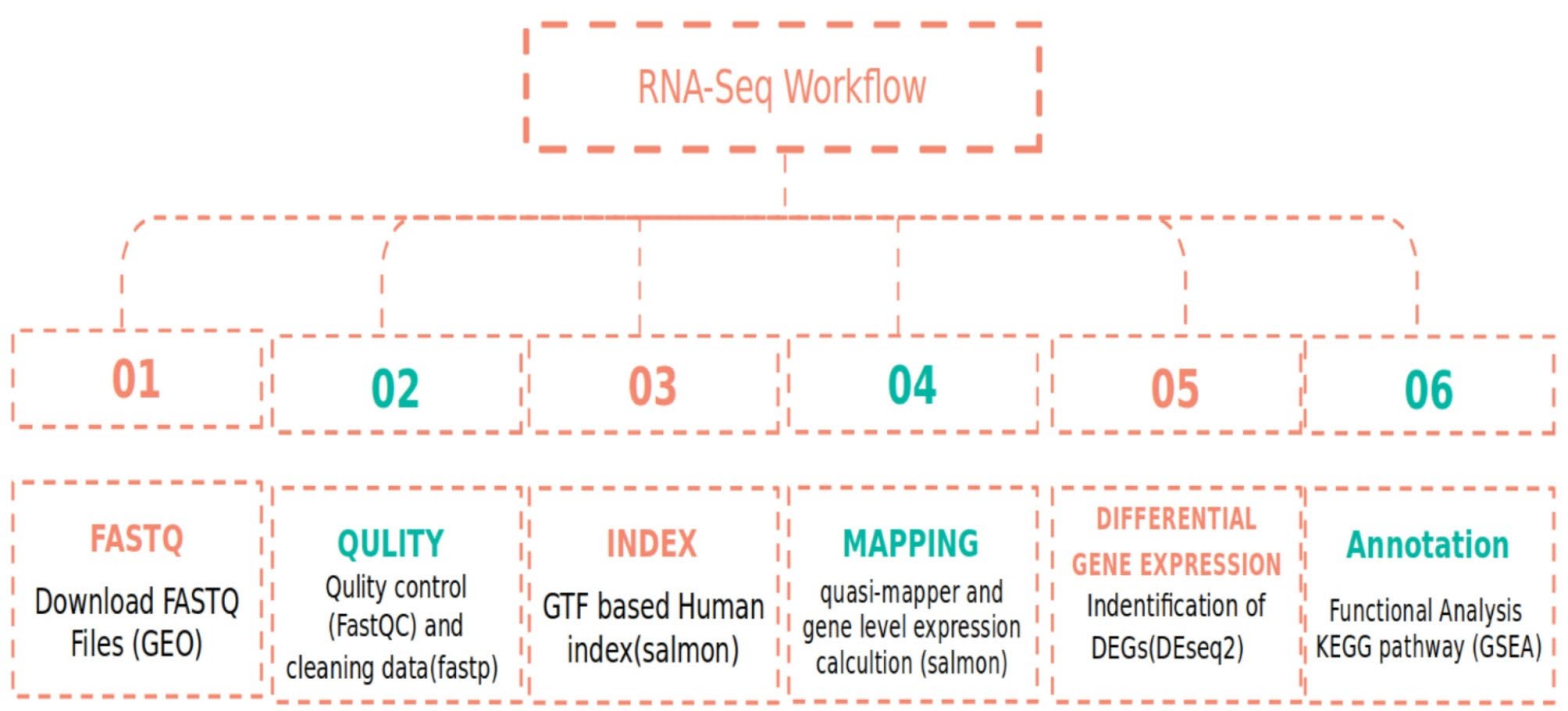
Workflow Diagram for the Conducted Analysis Study.

As shown in Fig. 7, For bulk RNA-Seq data, quality control and sequence alignment were firstly conducted using fastp^57^. The current human reference genome (GRCh38) was used and index were made by salmon. Gene expression was quantified using salmon quant^58^. Quality-controlled and trimming the transcript abundance from a single sample fastp (version 0.23.0). We used quality-controlled RNA-seq runs with parameters phred scores 15 and length required 20 to calculate the expression levels in each sample. We then used Salmon Trimmed Mean of M values (TMM) to normalize the expression levels across samples, after condensing the quantitative data of all samples into an expression matrix. after sequence alignment utilizing salmon index and the batch effect was eliminated using the R package ‘limma’. Genes with zero values in > 10% of the samples were excluded. Differentially expressed genes (DEGs) were identified by R package 'DESeq2′^59^.

### Targeting the probable roles of these common genes and associating them with Alzheimer’s disease pathways

To better understand the target genes’ biological function, we conducted Gene Ontology (GO) annotation and Kyoto Encyclopedia of Genes and Genomes (KEGG) pathway enrichment analyses using the Omics Share biological information cloud platform. The GO annotation included three components: biological process (BP), cellular component (CC), and molecular function (MF). Statistical significance was determined by a p-value threshold of less than 0.05. KEGG enriched and screened the top 20 items of the p-value.

## Conclusion

The research thus, identifies a significant association between post-COVID-19 syndrome and dysregulated pathways, including oxidative phosphorylation, inflammatory processes, and extracellular matrix-related mechanisms. Genes like ITGB5, CASP4, RYR2, MT-ND, and MT-CO hinder these pathways, impacting mitochondrial function and apoptosis regulation. Notably, ITGB5’s (Integrin beta) involvement in cell adhesion affects mitochondrial function, while CASP4 (Caspase 4) influences apoptosis and mitochondrial function. Furthermore, RYR2 (Ryanodine Receptor 2) dysfunction is linked to altered autophagic flux and neurodegenerative diseases like Alzheimer’s, impacting Ca^2+^ release. Retrograde nerve transmission suggests viral entry via peripheral nerves into the brain, with inflammation facilitating brain infection. Dysregulated ECM-receptor interaction and cytokine pathways contribute to BBB dysfunction. Overall, the study highlights the interconnectedness of pathways in COVID-19 recurrence, emphasizing the role of mitochondria, inflammation, and BBB integrity in post-infection complications. The significance of sustained systemic inflammation and blood–brain barrier (BBB) dysfunction in post-COVID-associated brain fog and its potential link to Alzheimer’s probability in the future is notable as the persistent inflammation, promoting brain fog condition creates a cognitive impairment condition in patients. Further research in this domain could provide insights into how BBB dysfunction or systemic inflammation contributes to cognitive impairment in COVID patients and functions as biomarkers. By elucidating the underlying mechanisms, therapeutic interventions could be developed to prevent or delay AD onset in susceptible individuals, including those with a history of COVID-19 infection.

## Electronic supplementary material

Below is the link to the electronic supplementary material.

Supplementary Material 1

## Data Availability

All data and materials used in this research are freely available and can be obtained upon request from the corresponding author.
