# Supplementary material for: Omics-based analysis of mitochondrial dysfunction and BBB integrity in post-COVID-19 sequelae
Source: Sci Rep. 2024 Dec 28;14:31016. doi: 10.1038/s41598-024-82180-6 (PMC11681072; doi:10.1038/s41598-024-82180-6)
Supplement: Supplementary file 1 — Supplementary Material 1 [file 41598_2024_82180_MOESM1_ESM.docx]

**Supplementary table 1:** List of accession numbers of control and COVID-19 infected cell transcriptome in the GEO database.

|  | **PRJNA1055552-GSE251849 (n=23)** | | |
| --- | --- | --- | --- |
| **No.** | **Sample Name** | **Group** | **Source** |
|  | GSM7989065 | COVID convalescent | PBMC |
|  | GSM7989064 |  |  |
|  | GSM7989063 |  |  |
|  | GSM7989062 |  |  |
|  | GSM7989061 |  |  |
|  | GSM7989060 | Healthy |  |
|  | GSM7989059 |  |  |
|  | GSM7989058 |  |  |
|  | GSM7989057 |  |  |
|  | GSM7989056 |  |  |
|  | GSM7989055 |  |  |
|  | GSM7989054 |  |  |
|  | GSM7989072 | Long COVID |  |
|  | GSM7989071 |  |  |
|  | GSM7989070 |  |  |
|  | GSM7989069 |  |  |
|  | GSM7989068 |  |  |
|  | GSM7989066 |  |  |
|  | GSM7989076 | Long COVID brain fog |  |
|  | GSM7989075 |  |  |
|  | GSM7989074 |  |  |
|  | GSM7989073 |  |  |
|  | GSM7989067 |  |  |
|  | **PRJNA745903-GSE179923(n=30)** | | |
|  | GSM5436983 | Blood_vessel | Blood_vessel_preparation |
|  | GSM5436983 |  |  |
|  | GSM5436985 |  |  |
|  | GSM5436985 |  |  |
|  | GSM5436987 |  |  |
|  | GSM5436987 |  |  |
|  | GSM5436977 | Mock | BCECs |
|  | GSM5436977 |  |  |
|  | GSM5436979 |  |  |
|  | GSM5436979 |  |  |
|  | GSM5436980 |  |  |
|  | GSM5436980 |  |  |
|  | GSM5436981 |  |  |
|  | GSM5436981 |  |  |
|  | GSM5436982 |  |  |
|  | GSM5436982 |  |  |
|  | GSM5436984 |  |  |
|  | GSM5436984 |  |  |
|  | GSM5436974 | SARS-CoV-2 |  |
|  | GSM5436974 |  |  |
|  | GSM5436975 |  |  |
|  | GSM5436975 |  |  |
|  | GSM5436976 |  |  |
|  | GSM5436976 |  |  |
|  | GSM5436978 |  |  |
|  | GSM5436978 |  |  |
|  | GSM5436986 |  |  |
|  | GSM5436986 |  |  |
|  | GSM5436988 |  |  |
|  | GSM5436988 |  |  |
